# Routine stool microscopy unmasking strongyloidiasis: a case report from Eastern Uttar Pradesh

**DOI:** 10.3389/fpara.2026.1892905

**Published:** 2026-07-29

**Authors:** Shalini Mann, Harsh Raj, Anjan Trivedi

**Affiliations:** 1 Department of Microbiology, Shri Gorakshnath Medical College Hospital and Research Centre (SGMCHRC) Mahayogi Gorakhnath University, Gorakhpur, Uttar Pradesh, India; 2 Department of Medicine, Shri Gorakshnath Medical College Hospital and Research Centre (SGMCHRC) Mahayogi Gorakhnath University, Gorakhpur, Uttar Pradesh, India

**Keywords:** chronic diarrhoea, Eastern Uttar Pradesh, eosinophilia, farmer, Gorakhpur, rhabditiform larva, stool microscopy, *Strongyloides stercoralis*

## Abstract

Strongyloidiasis is an intestinal parasitic disease caused by Strongyloides stercoralis, a soil-transmitted nematode. It is a common infection in Southeast Asia, including India, and is related to poor sanitation, rural residence, barefoot walking, and occupational exposure to soil. We report a case of a 71-year-old male farmer from rural Gorakhpur, Eastern Uttar Pradesh, who presented with intermittent watery diarrhoea for 2 years and tenesmus for 15 days. He was admitted with a diagnosis of fever of unknown origin. Routine stool microscopy revealed motile rhabditiform larvae of Strongyloides stercoralis, which were identified by their characteristic morphology, including a short buccal capsule and prominent genital primordium. Haematological examination revealed mild anaemia and eosinophilia. No other ova, cysts, or trophozoites were observed in the stool samples. The patient was treated with 200 µg/kg/day ivermectin for 2 days and showed clinical improvement. This case highlights the importance of routine stool microscopy in patients with chronic gastrointestinal complaints, especially in elderly patients from rural agricultural backgrounds

## Introduction

*Strongyloides stercoralis* is an intestinal nematode that is an important cause of soil-transmitted helminthic infections. Humans become infected by the penetration of the skin by infective filariform larvae in contaminated soil. The infection is related to tropical and subtropical climates, rural residence, poor sanitation, low socioeconomic status, walking barefoot, and frequent contact with contaminated soil.

The clinical spectrum of strongyloidiasis ranges from asymptomatic infection to gastrointestinal, pulmonary, dermatological, and disseminated diseases (Greaves et al., 2013). Gastrointestinal manifestations include abdominal pain, diarrhoea, altered bowel habits, malabsorption, bloating, and tenesmus. Eosinophilia can be observed, especially in chronic infections, but it can be variable.

Larval excretion in the stool may be intermittent, so diagnosis is often missed. Thus, stool examination continues to be a valuable and feasible diagnostic tool, particularly in resource-limited settings. Early diagnosis is clinically important because *S. stercoralis* can sustain infection through autoinfection, which may last for years. Untreated infections in the elderly or immunocompromised individuals may lead to hyperinfection syndrome or disseminated strongyloidiasis.

We report a case of intestinal strongyloidiasis diagnosed by routine stool microscopy in a 71-year-old farmer from rural Gorakhpur in Eastern Uttar Pradesh, India.

## Case presentation

We report a 71-year-old male patient from a village area of Gorakhpur district, Eastern Uttar Pradesh, India, who presented with intermittent watery diarrhoea, occurring 4–5 times/day, for the past 2 years. Fifteen days before admission, he developed tenesmus and was subsequently admitted with fever of unknown origin. He was a farmer and had regular contact with soil during agricultural activities.

He did not have any past medical history or known risk factors such as HIV, malignancy, organ transplantation, chronic kidney disease, corticosteroid use, chemotherapy or any other immune deficiency; therefore, he would be classified as immunocompetent. There were no other cases of gastrointestinal illness or parasitic infection diagnosed in family members. During his stay in the hospital, he had a diagnosis of symptomatic cholelithiasis and had an elective laparoscopic cholecystectomy after recovering from the presenting illness. Upon physical examination, the abdomen was examined and found to be soft and non-tender with no organomegaly or other significant abnormalities noted. Simultaneously, other tests were performed, and the results indicated mild anaemia, characterized by haemoglobin 11.7 g/dL and packed cell volume 34%. The total red blood cell count was 4.31 million/cumm. The red cell indices were as follows: MCV, 78.9 fL; MCH, 27.2 pg; MCHC, 34.5 g/dL; and RDW, 13.6%. The total leukocyte count was 5,500/cumm. In the differential leukocyte count, neutrophils accounted for 43.8%, lymphocytes for 39.4%, monocytes for 8.5%, eosinophils for 7.1% (an absolute eosinophil count of 391 cells/mm³), and basophils for 1.2%. Eosinophilia suggests a likely parasitic worm infection. Both liver and kidney function tests were within the normal range.

**Figure 1:**
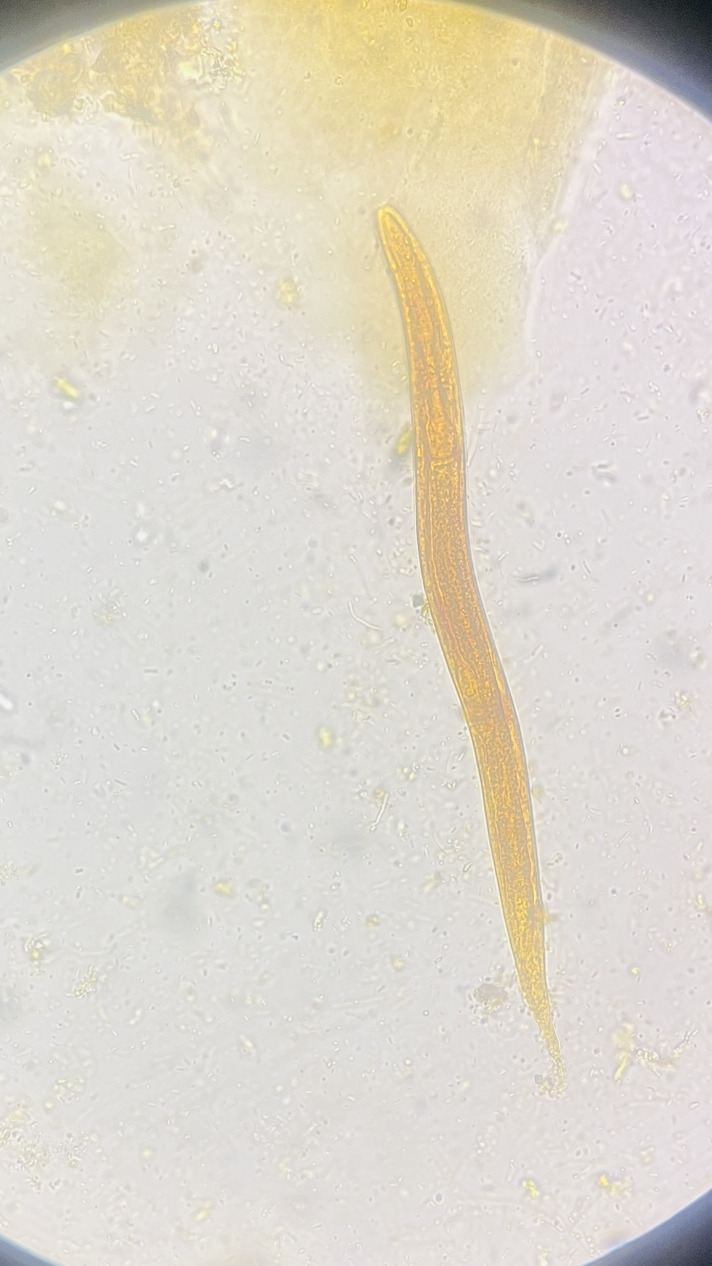
Rhabditiform larva of *Strongyloides stercoralis* observed in a iodine mount of the stool sample, showing a short buccal cavity and prominent genital primordium, characteristic features used for species identification.

A fresh stool specimen was sent to the Department of Microbiology for routine parasitological examination. The consistency of the stool was watery. Gross examination revealed no blood, mucus, adult worms, or proglottids. The stool was examined microscopically using saline and iodine wet mounts. Saline wet mount showed motile rhabditiform larvae which were identified as Strongyloides stercoralis based on the characteristic short buccal cavity and prominent genital primordium. Iodine wet mount helped in better visualization of the internal structure of the larvae (**Figure 1**). No other ova, cysts, or trophozoites were observed.

The patient was treated with oral ivermectin (200 µg/kg). By the end of 3 days after starting the treatment, the patient experienced clinical improvement, with no more fever, diarrhoea, and tenesmus, and he was discharged from the hospital in stable condition. Because the patient had no symptoms of respiratory infection or signs of hyperinfection syndrome, a sputum examination was not warranted, and therefore it was not performed. The patient underwent elective laparoscopic cholecystectomy for symptomatic cholelithiasis after his treatment; however, because he did not come back for parasitological follow-up after surgery, he will not have had a repeat stool examination, although he remains clinically asymptomatic.

## Discussion

Strongyloidiasis is an important but often underdiagnosed and neglected tropical disease caused by intestinal parasitic infection caused by *Strongyloides stercoralis* (WHO). It is commonly reported in tropical and subtropical regions and is associated with poor sanitation, low socioeconomic status, rural residence, and repeated exposure to contaminated soil. Farmers are at an increased risk because of frequent occupational contact with soil during agricultural activities. In the present case, the patient was a 71-year-old farmer from rural Gorakhpur, Eastern Utta Pradesh, with prolonged soil exposure was a likely risk factor for acquiring the infection. Similar risk factors have been described in earlier studies (Olsen et al., 2009; Krolewiecki et al., 2013).

The patient presented with chronic intermittent watery diarrhoea for 2 years and recent onset of tenesmus for 15 days. The clinical presentation of strongyloidiasis is variable and may range from asymptomatic infection to chronic gastrointestinal symptoms, pulmonary manifestations, and disseminated diseases. Chronic intestinal infection commonly presents with abdominal pain, diarrhoea, altered bowel habits, bloating, and malabsorption (Toledo et al., 2015). Tenesmus in this patient may be related to intestinal irritation or inflammation caused by the parasite.

In the present case, the diagnosis was made using routine stool microscopy, which revealed rhabditiform larvae of *S. stercoralis*. Unlike many other intestinal helminthic infections, *Strongyloides* infection is usually diagnosed by the detection of larvae rather than ova in the stool. The larvae were identified by characteristic morphology, including a short buccal capsule and prominent genital primordium. No other ova, cysts, or trophozoites were detected in the stool sample, supporting *S. stercoralis* as the significant parasite in this case.

The patient had mild eosinophilia with an absolute eosinophil count of 391 cells/mm³. Eosinophilia is frequently associated with helminthic infections and may provide clue in the diagnosis of chronic strongyloidiasis. However, eosinophilia may be variable, and its absence does not exclude the diagnosis (4 ).Serum IgE levels may also be elevated in patients with strongyloidiasis, although this was not evaluated in the present case.

Routine stool microscopy remains a simple, rapid, cost effective and useful diagnostic method, particularly in resource-limited settings. However, larval shedding in the stool may be intermittent; therefore, a single stool examination may result in false – negative finding which miss the diagnosis. Multiple stool examinations and concentration techniques can improve the diagnostic yield. Baermann concentration, agar plate culture, Harada-Mori filter paper culture, serological tests, and molecular methods may be used where available for improved detection (Requena-Méndez et al., 2013; Buonfrate et al., 2015).

Early diagnosis of strongyloidiasis is important because *S. stercoralis* has a unique ability to complete an autoinfection cycle within the host. This allows the parasite to persist for several years. Early diagnosis is therefore essential, particularly in elderly individuals and immunocompromised patients, in whom delayed diagnosis may result in hyperinfection syndrome or disseminated strongyloidiasis, both of which are associated with high mortality (Nutman, 2017). In the present case, the patient was immunocompetent, had no respiratory symptoms or other clinical evidence of hyperinfection syndrome, and therefore sputum examination was not clinically indicated. In elderly or immunocompromised patients, delayed diagnosis may increase the risk of hyperinfection syndrome and disseminated strongyloidiasis, which can be life-threatening (Nutman, 2017).

Ivermectin is the preferred treatment for strongyloidiasis. In the present case, the patient was treated with ivermectin at a dose of 200 µg/kg/day for 2 days and showed clinical improvement with complete resolution of gastrointestinal symptoms. Follow-up stool examination is recommended after treatment to document parasitological clearance, as autoinfection and intermittent larval shedding can lead to persistent infection (Henriquez-Camacho et al., 2016). Unfortunately, repeat stool examination could not be performed because the patient did not return for parasitological follow-up after subsequently undergoing elective laparoscopic cholecystectomy for symptomatic cholelithiasis. This represents a limitation of the present case report.

## Conclusion

This case highlights the significance of strongyloidiasis as one of the major yet commonly overlooked causes of chronic intermittent diarrhoea and tenesmus, particularly in individuals working outdoors such as farmers. The microscopic examination of stool specimens is a rapid and inexpensive test for diagnosing rhabditiform larvae of Strongyloides stercoralis, particularly in areas where there are limited resources available. Clinicians must be cautious about strongyloidiasis in patients presenting with persistent gastrointestinal symptoms, eosinophilia, and exposure to risk factors. Early and proper treatment of strongyloidiasis using ivermectin will prevent chronic infection and minimize the risk of developing serious complications.

### Limitations

The present report has some limitations. First, repeated stool examination could not be done because the patient did not come for follow-up examination after elective laparoscopic cholecystectomy; hence, clearance of the parasite could not be documented. Second, photomicrography was conducted without using a calibrated micrometry system, thus preventing the addition of an accurate scale bar. Nonetheless, the identification of the specimen was based on the known morphological characters such as short buccal cavity and prominent genital primordium. Last but not least, advanced diagnostic methods including serological or molecular techniques were unavailable, and the diagnosis was made using conventional microscopy of stool which is still useful in low-resource settings.

### Patient consent

In accordance with the institution’s policy, ethical approval has not been required for the single patient case report. Moreover, the patient has given written informed consent for the publication of clinical details exhibited in the article, along with the biomedical image associated with it. Patient’s identification has been removed from the material to ensure confidentiality.

## Data Availability

The original contributions presented in the study are included in the article/supplementary material. Further inquiries can be directed to the corresponding authors.

## References

[B1] Buonfrate D. Formenti F. Perandin F. Bisoffi Z. (2015). Novel approaches to the diagnosis of Strongyloides stercoralis infection. Clin. Microbiol. Infect. 21, 543–552. doi: 10.1016/j.cmi.2015.04.001 25887711

[B2] Greaves D. Coggle S. Pollard C. Aliyu S. H. Moore E. M. (2013). Strongyloides stercoralis infection. BMJ 347, f4610. doi: 10.1136/bmj.f4610 23900531

[B3] Henriquez-Camacho C. Gotuzzo E. Echevarria J. White A. C. Jr Terashima A. Samalvides F. . (2016). Ivermectin versus albendazole or thiabendazole for Strongyloides stercoralis infection. Cochrane Database Syst. Rev. 2016, CD007745. doi: 10.1002/14651858.CD007745.pub3 26778150 PMC4916931

[B4] Krolewiecki A. J. Lammie P. Jacobson J. Gabrielli A. F. Levecke B. Socias E. . (2013). A public health response against Strongyloides stercoralis: time to look at soil-transmitted helminthiasis in full. PloS Negl.Trop. Dis. 7, e2165. doi: 10.1371/journal.pntd.0002165 23675541 PMC3649958

[B5] Nutman T. B. (2017). Human infection with Strongyloides stercoralis and other related Strongyloides species. Parasitology 144, 263–273. doi: 10.1017/S0031182016000834 27181117 PMC5563389

[B6] Olsen A. van Lieshout L. Marti H. Polderman T. Polman K. Steinmann P. . (2009). Strongyloidiasis--the most neglected of the neglected tropical diseases? Trans. R. Soc Trop. Med. Hyg. 103, 967–972. doi: 10.1016/j.trstmh.2009.02.013 19328508

[B7] Requena-Méndez A. Chiodini P. Bisoffi Z. Buonfrate D. Gotuzzo E. Muñoz J. (2013). The laboratory diagnosis and follow up of strongyloidiasis: a systematic review. PloS Negl.Trop. Dis. 7, e2002. doi: 10.1371/journal.pntd.0002002 23350004 PMC3547839

[B8] Toledo R. Muñoz-Antoli C. Esteban J. G. (2015). Strongyloidiasis with emphasis on human infections and its different clinical forms. Adv. Parasitol. 88, 165–241. doi: 10.1016/bs.apar.2015.02.005 25911368

[B9] WHO Strongyloidiasis. Available online at: http://www.who.int/neglected_diseases/diseases/strongyloidiasis (Accessed July 18, 2026).

